# RAD51 inhibition in triple negative breast cancer cells is challenged by compensatory survival signaling and requires rational combination therapy

**DOI:** 10.18632/oncotarget.11065

**Published:** 2016-08-05

**Authors:** Adrian P. Wiegmans, Mariska Miranda, Shu Wen Wen, Fares Al-Ejeh, Andreas Möller

**Affiliations:** ^1^ Tumor Microenvironment Laboratory, QIMR Berghofer, Herston Rd, Herston QLD 4006, Australia; ^2^ Personalized Medicine Laboratory, QIMR Berghofer, Herston Rd, Herston QLD 4006, Australia; ^3^ School of Medicine, University of Queensland, Brisbane, QLD 4072, Australia

**Keywords:** RAD51, kinome, triple negative breast cancer, p38MAP Kinase, targeted therapy

## Abstract

The molecular rationale to induce synthetic lethality, by targeting defective homologous recombination repair in triple negative breast cancer (TNBC), has proven to have several shortcomings. Not meeting the expected minimal outcomes in clinical trials has highlighted common clinical resistance mechanisms including; increased expression of the target gene PARP1, increased expression or reversion mutation of BRCA1, or up-regulation of the compensatory homologous recombination protein RAD51. Indeed, RAD51 has been demonstrated to be an alternative synthetic lethal target in BRCA1-mutated cancers. To overcome selective pressure on DNA repair pathways, we examined new potential targets within TNBC that demonstrate synthetic lethality in association with RAD51 depletion. We confirmed complementary targets of PARP1/2 and DNA-PK as well as a new synthetic lethality combination with p38. p38 is considered a relevant target in breast cancer, as it has been implicated in resistance to chemotherapy, including tamoxifen. We show that the combination of targeting RAD51 and p38 inhibits cell proliferation both *in vitro* and *in vivo*, which was further enhanced by targeting of PARP1. Analysis of the molecular mechanisms revealed that depletion of RAD51 increased ERK1/2 and p38 signaling. Our results highlight a potential compensatory mechanism via p38 that limits DNA targeted therapy.

## INTRODUCTION

Triple negative breast cancer (TNBC), lacking the expression of estrogen receptor, progesterone receptor and human EGF receptor-2, represents an especially aggressive and hard to treat subtype. Chemotherapy is the only standard treatment available for TNBC patients to date [1]. New targets for TNBC have been proposed to focus on oncogenic lesions and metabolic abnormalities that support the aggressive phenotype [2]. These drivers include; oncogene mutation, tumor suppressor loss, mutation or overexpression of DNA repair proteins and enhanced survival via kinase signaling [2].

Gene expression profiling identified that approximately 80% of sporadic TNBC are “basal-like”, with aberrations mimicking defective homologous recombination (HR) DNA repair and displaying BRCA-ness [3]. BRCA-ness creates a shift from HR, which is dysfunctional, towards alternative DNA repair mechanisms, including those regulated by poly(ADP-ribose)polymerases (PARPs). This has provided the rationale for testing PARP inhibitors in TNBC to create synthetic lethality, the mechanism by which the targeting of two genes is lethal, while individually they are not. However, targeting PARP1/2 has had limited clinical success [4]. PARP inhibition on a BRCA1 background is readily overcome by 53BP1 loss, BRCA1 reversion and upregulation of RAD51, all compensatory mechanisms of DNA repair [5].

In search of a novel and rational therapeutic combination for TNBC, several studies identified RAD51 as target in synthetic lethal screens for PARP inhibition in breast cancer [6–10]. RAD51 has been associated with compensating for functional BRCA1 loss [11] and we have previously published that RAD51 can support metastatic progression of TNBC [12]. We suggested that the pressure on the DNA repair pathways induced by the targeting of PARPi on a BRCAness background forces compensation by RAD51, providing a rationale for its targeting [13]. However, targeting of RAD51 has been shown to increase kinase signaling, suggesting additional mechanisms contributing to loss of synthetic lethality [14, 15]. Importantly, kinase signaling in TNBC is complex and characterized by several rewiring events as compensatory mechanisms for treatment resistance [16, 17]. We recently carried out antibody-based microarray analysis to study protein and phosphoprotein expression in breast cancer, including TNBC. We described that 50% of TNBC lines or cases have very complex kinome profiles, which may explain their resistance to chemotherapy as well as targeted therapies [18].

Following our previous studies on RAD51 [12, 13] and kinome profiling in TNBC [18], in this study we now investigate whether RAD51 inhibition synergizes with inhibitors of kinases that are activated in TNBC. We find that RAD51 inhibition synergizes with several inhibitors, most notably PARP and p38 inhibitors. This synergy is characterized by rewiring of phosphorylation patterns after RAD51 inhibition, which are attenuated by combining with both PARP and p38 inhibition *in vitro* and *in vivo*. We propose that kinome rewiring limits the efficacy of targeting RAD51 and that rational targeted combination therapy is required to achieve effective therapy against TNBC.

## RESULTS

### The effect of RAD51 depletion on the sensitivity of TNBC cells to inhibitors of the DNA-damage response and kinase signaling pathways

Our previous studies showed that depletion of RAD51 limits metastatic progression of TNBC [12], and we suggested that the pressure on the DNA repair pathways induced by the targeting of PARP may force compensation through RAD51 [13], thus rationalizing targeting RAD51 in this setting. Since the targeting of RAD51 has been previously shown to increase kinase signaling [14, 15], we asked whether the combined inhibition of RAD51 and kinase signaling could be at least additive. To this end, we utilized our previous kinome profiling data to identify several activated kinases and overexpressed proteins in TNBC primary tumors and cell lines [18]. These include HSP90, PLCγ, PDK, EGFR, MEK, DNA-PK, GSK3β, PKC, PYK2, PAK, IGF-1R, p38, ERK5, S6K and LIG4. In addition, we also included PARP1, ATM and CHK1 inhibition, based on our hypothesis that targeting of complementary DNA-damage response (DDR) pathways would be most effective in combination with RAD51 depletion.

Using two different siRNA targeting RAD51 in three TNBC cell lines (MDA-MB-231, MDA-MB-436 and PMC42-ET), in the presence of drugs targeting the selected 18 proteins, the effectiveness of the combination was examined using a ratio of IC50 values comparing RAD51 knockdown to scrambled control lines. In line with our hypothesis that targeting complementary DDR pathways should be effective with RAD51 depletion, we found that PARP1 inhibition was most effective in RAD51 knockdown cell lines (average of knockdown over scrambled ratio IC50′s is 0.39 across all cell lines, Table 1). This finding is consistent with several reported synthetic lethality screens involving PARP inhibitors [8–10]. Inhibition of the DNA-dependent protein kinase (DNA-PK) and RAD51 depletion was also a potent combination (average 0.67, Table 1). Of the remaining drugs, 4 were additive in two of the 3 lines (inhibitors of LIG4, HSP90, MEK and EGFR), while 4 were additive across all 3 cell lines (p38, S6K, PAK, PYK2). Interestingly, the inhibition of p38 was more potent than DNA-PK inhibition (p=0.041, Table 1). p38 signaling by constitutively active MKK6 (MKK6E) has been shown to increase level of RAD51 protein, improving its stability without altering the level of RAD51 mRNA [19]. This may explain the effect of combining RAD51 depletion with p38 inhibition.

**Table 1:** Ratio of IC50s for RAD51 knockdown and drug targets in three TNBC cell lines

IC50 Target	MDA-MB-231			MDA-MB-436			PMC42-ET			Ave	*p* Value
	SCR	RAD51	Ratio	SCR	RAD51	Ratio	SCR	RAD51	Ratio		
PARP1	32.26	9.86	0.3056	6.92	3.22	0.4653	35.01	13.73	0.3922	**0.3877**	**0.028**
P38	16.13	12.7	0.7874	12.92	7.877	0.6097	19.69	10.43	0.5297	**0.6422**	**0.041**
DNA-PK	1.02	0.69	0.6330	2.02	1.6	0.7921	1.89	1.1	0.5820	**0.6690**	0.051
S6K	9.38	3.965	0.4227	4.838	2.944	0.6085	1.587	1.623	1.0227	**0.6846**	0.268
ATM	20.25	9.54	0.4711	33.94	25.07	0.7386	21.94	21.94	1.010403R3	**0.7366**	0.187
PAK	20.39	15.08	0.7396	13.1	11.85	0.9046	16.95	11.9	0.7021	**0.7821**	0.098
LIG4	19.01	19.4	1.0205	20.07	15.51	0.7728	12.94	7.716	0.5963	**0.7965**	0.219
CHK1	0.09	0.08	0.8889	0.03	0.03	1.010403R3	0.15	0.1	0.6667	**0.8519**	0.312
PYK2	0.098	0.088	0.9025	20.96	16	0.9025	13.17	10.55	0.9025	**0.9025**	0.219
HSP90	0.14	0.12	0.8330	0.1698	0.1816	1.0695	0.21	0.20	0.9393	**0.9472**	0.52
GSK3	12.13	13.12	1.0816	20.55	17.22	0.8380	ND	ND	ND	**0.9598**	0.684
DNA-PK(2)	0.097	0.094	0.9678	0.096	0.093	0.9676	0.094	0.089	0.9501	**0.9619**	**0.032**
MEK (2)	13.23	16.89	1.2766	17.49	9.122	0.5216	14.88	17.69	1.1888	**0.9957**	0.885
EGFR	0.21	0.19	0.8984	0.1705	0.2248	1.3185	0.20	0.17	0.8485	**1.0218**	0.986
PDK	12.58	18.38	1.4610	14.58	15.04	1.0316	18.08	15.05	0.8324	**1.1083**	0.716
PKC	15.41	13.75	0.8923	17.26	23.82	1.3801	11.67	12.48	1.0694	**1.1139**	0.516
MEK	0.5591	0.7839	1.4021	0.097	0.091	0.9986	0.098	0.093	0.9464	**1.1157**	0.451
PLC	16.13	22.70	1.4073	16.13	18.27	1.1327	20.55	16.72	0.8136	**1.1179**	0.643
IGF-1R	9.54	17.97	1.8836	14.51	10.34	0.7126	ND	ND	ND	**1.2981**	0.792
ERK5	4.151	8.943	2.1544	5.018	6.049	1.2055	3.041	2.25	0.7399	**1.3666**	0.415

The IC50 values in μM were established for individual drugs in three TNBC cell lines that express high levels of RAD51. They are ranked based on the difference in IC50 values across the three cell lines for RAD51 depletion with a drug versus no RAD51 depletion. A score below 1.0 was deemed to be additive while above 1.0 was antagonistic.

### RAD51 inhibition in combination with p38 and PARP inhibition

**Figure 1:**
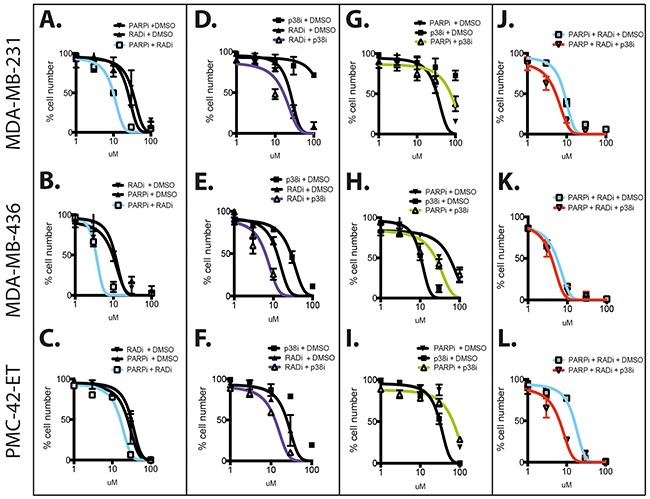
Combination of RAD51, PARP and p38 inhibitors against TNBC cell lines The three TNBC cell lines MDA-MB-231 (top row), MDA-MB-436 (middle row) and PMC42-ET (bottom row) were used for dose response studies using three molecular inhibitors targeting RAD51 (B02, RAD51i), PARP (ABT-888, PARPi) and p38 (LY2228820, p38i). Dose curves for the single drugs were performed using 0-100 μM escalating doses. The combinations were carried out as follows: **A–C.** RAD51i escalating doses 0-100 μM with 2.5 μM of PARPi; **D–F.** RAD51i escalating doses 0-100 μM with 10 μM of p38i; **G–I.** p38i escalating doses 0-100 μM with 2.5 μM of PARPi **J–L.** RAD51i escalating doses 0-100 μM with 2.5 μM PARPi alone or with 10 μM of p38i. All experiments were performed over 72 hours in triplicate and graphs represent line of best-fit non-linear regression +/−SEM.

Next, we validated our findings using the RAD51 inhibitor B02 [20]. We confirmed the activity of B02 as a potent inhibition of RAD51 foci formation after irradiation (Supplementary Figure S1B). We also confirmed the activity of the PARPi ABT-888, which showed concentration-dependent PARP inhibition of protein PARylation (Supplementary Figure S1A). The activity of the p38 inhibitor LY2228820 (p38i) demonstrated restricted phosphorylation of MK2 (Supplementary Figure S1C), a p38 downstream target [21]. In line with the combination of RAD51 depletion and PARP inhibition, each of the three TNBC cell lines displayed a decrease in IC50 when targeted with the RAD51 inhibitor B02 (RAD51i) in combination with PARPi (2.5 μM ABT-888) compared to PARPi alone (Figures 1A-1C). We also confirmed that the combination of RAD51i and p38i is more potent than each drug individually (Figures 1D-1F). The specific effects of combining RAD51i/PARPi or RAD51i/p38i were supported by the lack of benefit from combining PARPi and p38i (Figure 1G-1I). Given the role of p38 signaling in the stabilization of RAD51 protein (16), we questioned whether p38 inhibition could further enhance the effect of the combination of RAD51 and PARP inhibition. Indeed, p38i significantly potentiated RAD51i/PARPi combination in the three TNBC cells lines (Figures 1J-1L), suggesting that p38 signaling may be an escape mechanism from the combined inhibition of RAD51 and PARP.

### Targeting of RAD51 activates p38 signaling via ERK1/2

**Figure 2:**
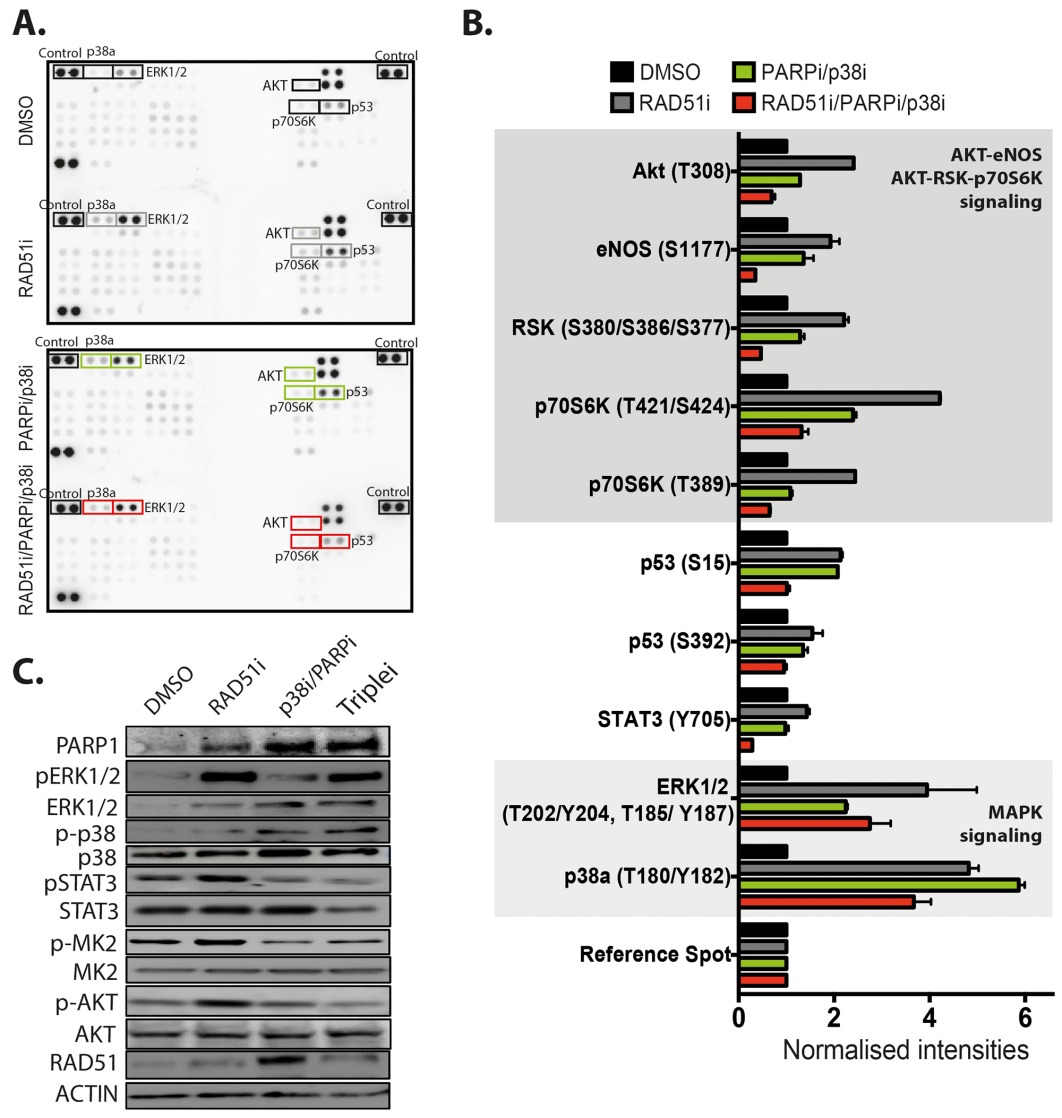
Targeting of RAD51 activates several signaling pathways but attenuated by combining with PARP and p38 inhibition **A.** The Human Phospho-Kinase arrays (R&D Systems) were probed with MDA-MB-231 lysate samples that had been treated for 72 hours; samples used are labeled (from top to bottom): (1) DMSO treatment, (2) 10 μM RAD51i, (3) 2.5 μM PARPi/10 μM p38i, and (4) 10 μM RAD51i /2.5 μM PARPi/10 μM p38i triple combination. Highlighted dots represent a significant change in signal over DMSO treated controls. The corners are positive control blots for quantification. **B.** Quantitation of spot intensity was standardized for cells treated with DMSO and plotted as normalized intensity. Several kinases displayed greater than 2 fold increase in phosphorylation compared to references. Shading represents grouping based on pathway signaling. **C.** Protein expression and changes in phosphorylation of ERK1/2, p38, STAT3, MK-2 (p38 target) and AKT were confirmed by western blotting.

To investigate the mechanism of how p38 affects RAD51 inhibition, we examined the kinase signaling network in MDA-MB-231 cells in response to RAD51 inhibition (RAD51i), dual PARP and p38 inhibition (PARPi/p38i), and the triple combination (RAD51i/PARPi/p38i). We hypothesized that key kinases might be induced by RAD51 inhibition and/or dual PARP and p38 inhibition, but are significantly reduced by triple combination. We found significantly increased signaling in 14 of 44 kinases under these treatment conditions compared with DMSO controls (Figure 2A). RAD51i and the dual combination of PARPi/p38i significantly induced the MAPK signaling axis, particularly ERK1/2 and p38 (Figure 2B). Although the triple combination, RAD51i/PARPi/p38i, attenuated this induction, the phosphorylation of ERK1/2 and p38 remained higher than in the vehicle control (Figure 2B and 2C). It is vital to note that the p38 inhibitor LY2228820 does not affect the phosphorylation of p38 but inhibits its activity [21, 22], which is determined by the phosphorylation of MK2. The RAD51 inhibitor significantly increased signaling via p38 with increased phosphorylation of MK2 (Figure 2C). The use of the p38 inhibitor in the triple combination did not significantly attenuate the phosphorylation of p38, but inhibited its activity as expected as judged by reduced MK2 phosphorylation (Figure 2C). Moreover, while the AKT-RSK-p70S6K and AKT-eNOS signaling axes were induced by RAD51i, these axes were significantly reduced by the RAD51i/PARPi/p38i triple combination (Figure 2B). This triple combination reduced pSTAT3 (Y705) under the baseline level of vehicle control, and reverted p53, S15 and S392 phosphorylations to the baseline levels. Collectively, our results show that the triple combination led to the inhibition of the induced p38 activity, AKT signaling pathways, STAT3 phosphorylation and p53 phosphorylation, which were induced by either RAD51 inhibition and/or the dual PARP/p38 inhibition. This significant reduction of survival signaling pathways explains the effectiveness of this combination *in vitro*.

### Effects of targeting RAD51, PARP and p38 *in vitro* and *in vivo*

**Figure 3:**
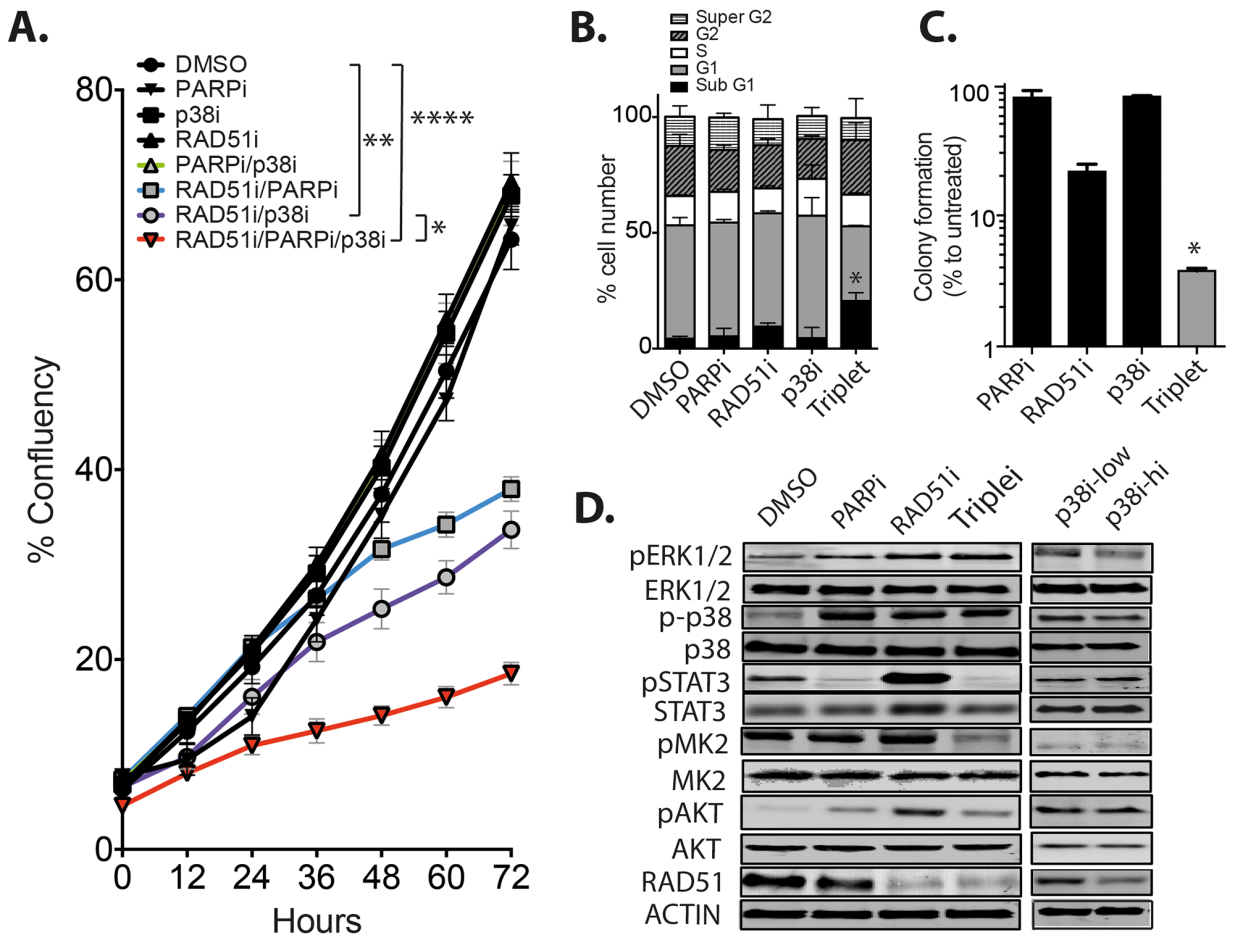
The combination of RAD51/PARP/p38 inhibition retards TNBC growth *in vitro* **A.** MDA-MB-231 cells were incubated in the presence of single, dual or triple drug combinations using 10 μM RAD51i, 10 μM p38i and 2.5 μM PARPi and cell growth followed over 72 hours. Results are average percentage confluency of the well (*p<0.05, **p<0.01, ****p<0.001). **B.** Cell cycle analysis of MDA-MB-231 cells treated with each inhibitor individually and in combination *p<0.05. **C.** Long term 14-day colony assays were performed on treated MDA-MB-231 and expressed as percentage of DMSO treated cells *p<0.05. **D.** Expression of key signaling proteins were examined after single drug treatment or the triple combination, p38i was utilized at low concentration of 10 μM as used in the combination therapies and at 100 μM high dose to observe any changes in expression.

To gain understanding of the consequences of the RAD51i/PARPi/p38i triple combination, we carried out *in vitro* and *in vivo* studies using the MDA-MB-231 TNBC cells. The triple combination significantly retarded short-term proliferation of MDA-MB-231 cells compared to vehicle and single drug controls (p<0.001), as well as the two-agent combinations (p<0.008, Figure 3A). Next, we examined the cell cycle profile in response to individual drugs and the triple combination at 72 hours post-treatment. There was no change in the cell cycle profiles of MDA-MB-231 cells in response to any of the individual inhibitors at the selected time point (Figure 3B). In contrast, the triple combination induced significant cell death compared to untreated cells with enhanced sub-G1 population (p=0.05, Figure 3B). The increased apoptosis in the triple combination explains the reduced short-term proliferation (Figure 3A) and the significant reduction of long-term colony formation (p=0.024, Figure 3C). Protein analysis revealed that drug treatment increased p38 and ERK1/2 phosphorylation, except for PARPi treated cells, which retained low ERK1/2 signaling, while RAD51i treatment induced pSTAT3 signaling, in agreement with our earlier observations (Figure 2A). p38 signaling to its downstream target MK2 was significantly reduced in the p38 targeting and triple treatment groups, while pAKT signaling was only enhanced in the RAD51i treated group (Figure 3D). In MDA-MB-436 cells that harbor a pathogenic BRCA1 5396+1G>A mutation, we observed an expected G2 arrest in response to PARPi and the triple combination, with a corresponding increase in the polyploidy population (Supplementary Figure S2A). The treatments also correlated with potent inhibition of colony formation (p=0.027, Supplementary Figure S2B) with the remaining resistant cells retaining a normal cell cycle profile comparable to control cells (Supplementary Figure S2C). This suggests that the G2 arrest and polyploid population (presumably due to mitotic slippage) is another mechanism of response to the triple combination. Protein analysis of MDA-MB-436 cells also showed an induction of the phosphorylated form of p38, but its activity was reduced by the p38 inhibitor significantly in the triple combination, as judged by MK2 phosphorylation (Supplementary Figure S2D). Interestingly, total and phosphorylated ERK1/2 protein was significantly reduced under all conditions in the MDA-MB-436 (Supplementary Figure S2D), in contrast to its increased phosphorylation in the MDA-MB-231 cells.

**Figure 4:**
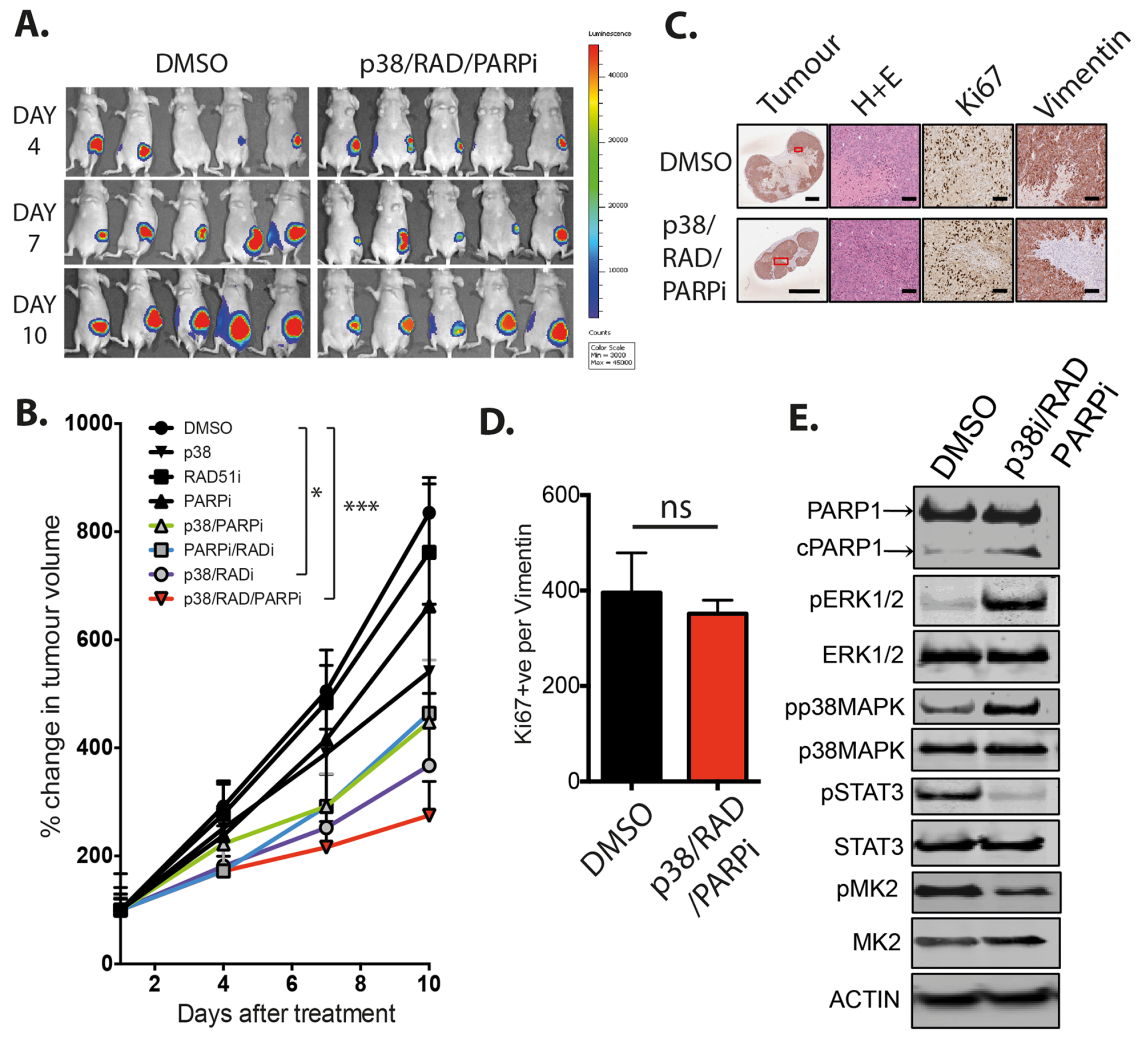
RAD51, PARP and p38 combined inhibition *in vivo* **A.** MDA-MB-231 xenografts were monitored by bioluminescence (luciferin) imaging, DMSO and triple therapy cohorts are represented. **B.** Tumor burden plotted as percentage change in volume for all single arm controls, double combinations and triple therapy combination compared to days after treatment with all three drugs. *p<0.05, ***p<0.005. **C.** Histological analysis for overall size, cellularity, proliferation and vimentin expression of DMSO and triple treated tumors (Scale bar =100 μm). **D.** Quantification of human proliferating cells (Ki67^+^, Vimentin^+^). **E.** Protein expression and signaling analysis of day 10 *ex vivo* DMSO and triple combination treated tumors.

To illustrate the effect of targeting RAD51, PARPi and p38, and the resulting signaling beyond *in vitro* studies, we examined the triple combination against MDA-MB-231 tumors *in vivo* (Figure 4A). The use of inhibitors against the individual targets did not significantly reduce primary tumor growth of orthotopic MDA-MB-231 mammary fat pad xenografts (Figure 4B). Of the dual combination therapies, RAD51i and p38i was the most effective compared to control cohort, with significant inhibition of tumor growth after 10 days of treatment (Figure 4B, p=0.019), in agreement with our *in vitro* results (Figure 3A). The triple combination inhibited tumor growth most significantly (Figure 4B and S3A, p=0.002). Reduced tumor burden was confirmed by assessing *ex vivo* mammary tumors (Figure 4C). At the ethically allowed maximal tumor size for the control cohort, there was no difference between treated and untreated samples in cancer cell proliferation (Figure 4D). Of note, DMSO control tumor Ki67^+^ number may have been limited by lack of blood supply to the inner mass. We further examined whether the tumors in the triple combination had sustained alterations in signaling that corroborate our *in vitro* findings. Indeed, *ex vivo* immunoblotting displayed induction of ERK1/2 and p38 and a decrease in STAT3 and MK2 phosphorylation in the triple treatment group (Figure 4E), associated with increased apoptosis judged by PARP cleavage (Figure 4E). As RAD51 is a key protein involved in the recombination of both B and T cell receptors, there is a general suggestion that therapeutically targeting of RAD51 in a combination therapy has the potential to induce myelosuppression [23]. Although we utilized immune-compromised nu/nu mice, they still retain functional immune cells except for T-cells, and we were able to detect no significant reduction in whole white blood cell count and lymphocytes in response to the triple combination, which all stayed within normal parameters (Supplementary Figure S3) [24–26]. Taken together, our *in vivo* studies confirmed our findings that p38 inhibition potentiates the cytotoxic effect of RAD51 inhibition alone and more so when combined with PARP inhibition in triple combination. Similar to our *in vitro* studies, the strong effect of the triple combination can be explained by the inhibition of survival signaling pathways, which are induced by the single or dual treatments.

### The rationale for targeting p38 with RAD51 is driven by signaling rather than changes in transcription of key proteins

**Figure 5:**
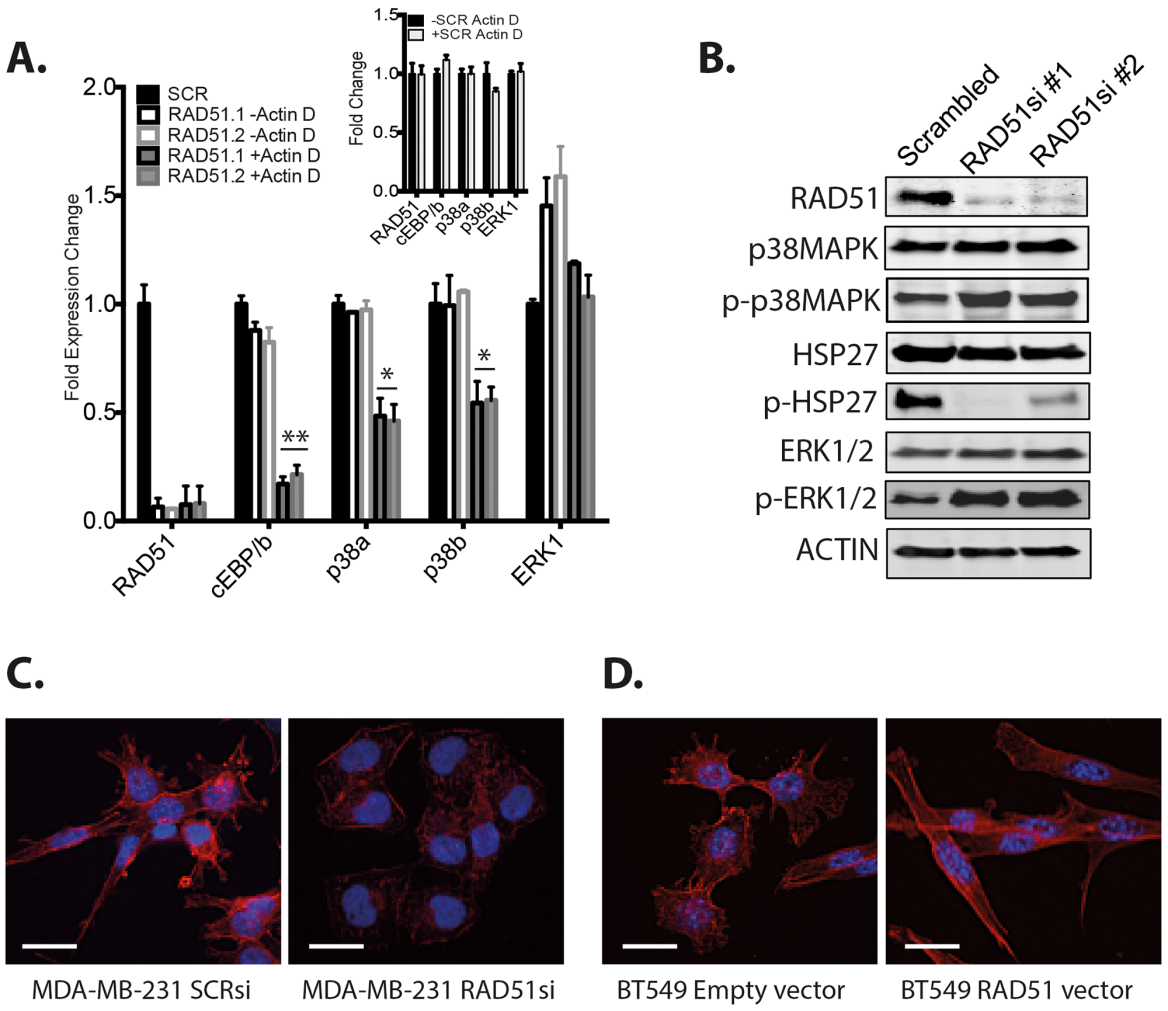
Analysis of p38 pathway signaling **A.** MDA-MB-231 cells were depleted of RAD51 and analyzed for expression of the specified genes in the presence or absence of the transcriptional inhibitor actinomycin D. Plots represent the fold expression change over GAPDH and are the average of triplicate experiments +/−SEM. (**A-Insert**) Controls for non-targeting siRNA +/− actinomycin D. **B.** Expression of the specified proteins and phosphoproteins examined after RAD51 depletion with two different siRNA. **C.** Immunofluorescence of MDA-MB-231 cells depleted of RAD51 and stained for morphology. **D.** Immunofluorescence of BT549 cells engineered for RAD51 overexpression and stained for morphology (Scale bar =100 μm).

The rationale behind combining RAD51 depletion and PARP1 inhibition is a well-defined set of mechanisms that coordinate inhibition of concordant DNA repair pathways [6–10]. The mechanisms by which RAD51 depletion combines with p38 inhibition are speculative. Previously, we found that RAD51 can bind c/EBPβ and influence gene expression in MDA-MB-231 cells [12]. Our data here suggest that RAD51 depletion or inhibition induces signaling in the p38 pathway. The significant induction of the transcriptional factor STAT3 suggested a possible transduction of signal from DNA damage to modulate transcription of specific genes [27]. To examine whether this signaling was related to changes in expression, we used the transcriptional repressor actinomycin D. Upon depletion of RAD51, compared to non-targeted controls (Figure 5A-insert), there was an associated depletion of *c/EBPβ, p38-alpha* and *p38-beta* transcript (Figure 5A, p<0.01, p<0.05), but not the *p38-delta* transcript (Supplementary Figure S4A). *p38-alpha* and *p38-beta* transcripts were also reduced in response to c/EBPβ depletion (Supplementary Figure S4B). This suggests that in the absence of the RAD51-c/EBPβ complex and active transcription, there is reduced expression or stability of these transcripts. Despite this interesting transcriptional regulation of *p38-alpha* and *p38-beta,* it is noteworthy that p38 protein levels after RAD51 targeting *in vitro/in vivo* or depletion with siRNA, were never down regulated (shown in Figure 5B).

Altogether, our transcriptional studies here support the notion that the increased phosphorylation of p38 is a *bona fide* signaling effect after RAD51 depletion, since p38 mRNA (Figure 5A) and protein (Figure 5B) did not increase even while transcription was active, or reduced when transcription was blocked with actinomycin D. Our initial drug screen supports this conclusion where the p38 inhibitor, but not MEK1 inhibitors, had an additive effect with RAD51 depletion, as p38 phosphorylation was increased as a possible compensatory mechanism due to the stress DNA repair inhibition.

### The targeting of p38 and RAD51 mediates additional anti-tumor effects via HSP27

The loss of HSP27 phosphorylation after RAD51 depletion (Figure 5B) provides further support to our conclusion. p38-mediated phosphorylation of HSP27 via MK2 has been demonstrated to regulate alpha-actin turnover [28]. The dephosphorylated form of HSP27 caps the barbed ends of actin filaments, while the phosphorylated form stabilizes long unbranched actin filaments [28]. This phenomenon was observed in morphological cell changes in response to RAD51 expression levels that correlate with HSP27 phosphorylation status, with contracted morphology after RAD51 depletion (Figure 5C) and elongated morphology with RAD51 overexpression (Figure 5D). This observation supports that p38 signaling plays a strong role during RAD51 modulation and further explains the benefit from combining p38 inhibition with RAD51 inhibition alone or when combined with PARP inhibition. The role for HSP27 phosphorylation in association with RAD51 in tumor progression is part of ongoing investigations.

## DISCUSSION

Therapies utilizing synthetic lethality with PARPi have been plagued by mechanisms of resistance. We show that by inducing synthetic lethality with PARPi and RAD51i, the addition of targeting p38 induces TNBC cell death *in vitro* and *in vivo*. p38 signaling effects associated with RAD51-mediated DNA repair is highly context-dependent, with both DNA-repair dependent and independent mechanisms reported [19, 29]. In contrast to our findings, several studies identified a role for p38 in the activation of Chk1 supporting G2 cell cycle arrest checkpoint assisting ATR signaling and DNA repair [30, 31]. We did not observe a G2 arrest under any single drug conditions except for the well-documented synthetic lethality of targeting PARP in MDA-MB-436. Previous studies also showed the inhibition of p38 activity had no effect on Chk1 activation and DNA repair [30], however, p38 was essential for survival through the expression of prosurvival apoptotic proteins [30]. This implies DNA repair-independent p38 survival activity, via changes in transcription. However, in the cancer setting, p38 can be essential for survival as a sensor for replicative stress, with loss of its downstream target MK2 protecting cells through a DNA-repair dependent activity [32]. Loss of MK2 signaling was observed in MDA-MB-231 cells upon treatment with the p38i/PARPi combination with concordant induction of RAD51 protein expression levels. This mechanism is contradicted by the observation that inhibition of p38 activation enhanced RAD51 protein instability, sensitizing cells to DNA damage [19]. We confirm reduced RAD51 expression observed with high dose p38 inhibitor, however, this was ten times the concentration required for *in vivo* therapy response. The targeting of p38 did result in loss of MK2 signaling, and the additional targeting RAD51 and PARP1 was able to overcome potentially enhanced DNA repair signaling. Therefore the targeting of p38 may protect cells from DNA damage, but is dependent upon a functional DNA repair pathway.

**Figure 6:**
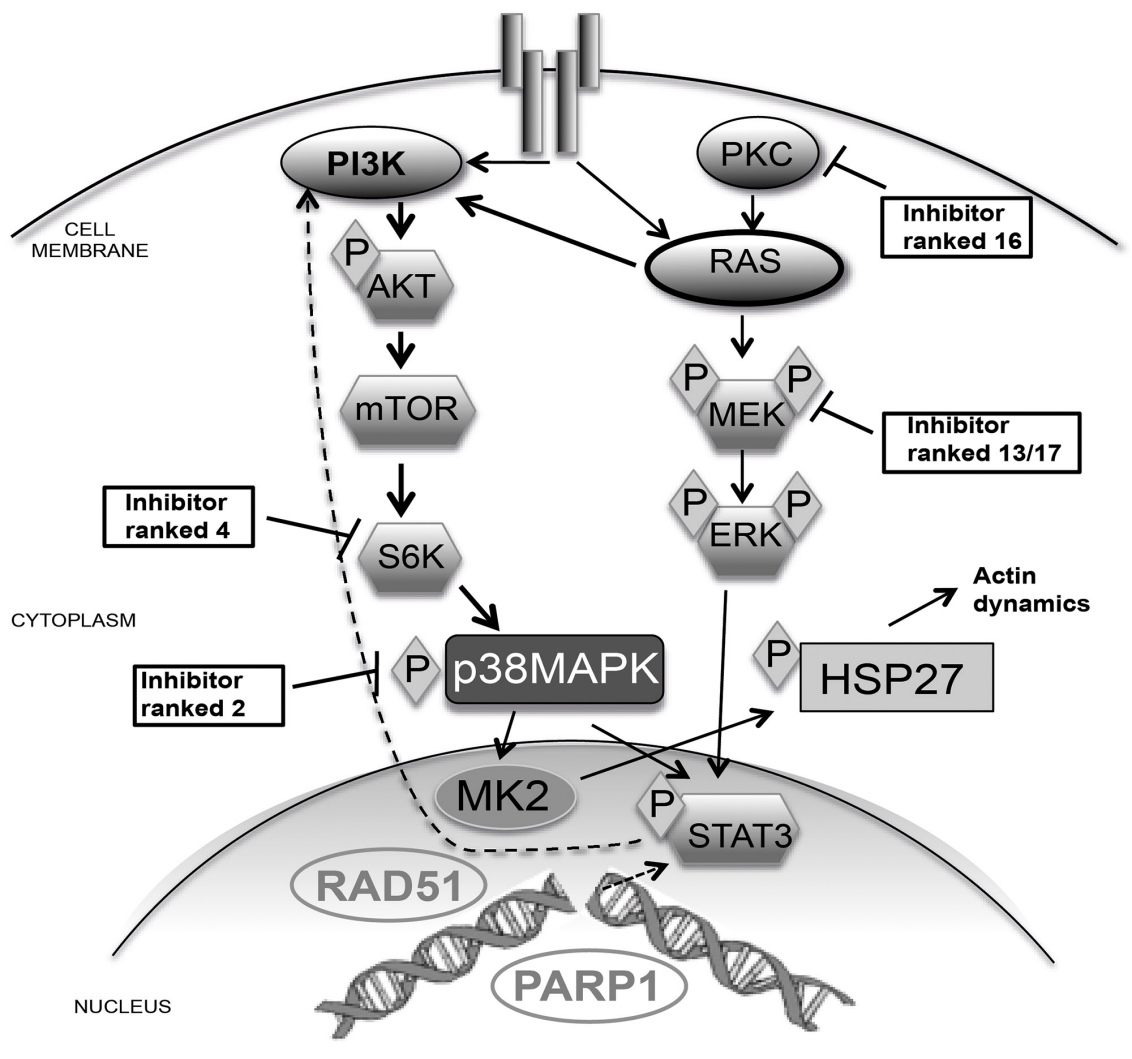
Schematic of p38 pathway signaling The schematic shows the signaling cascade from a receptor tyrosine kinase to the nucleus. We have highlighted some of the key proteins that were directly inhibited by small molecules and where they ranked in order of efficacy with RAD51 depletion. Due to a K-RAS mutation in MDA-MB-231 cells PI3K is highly active, however RAD51 depletion resulted in ERK1/2 activation. This is why the PKC and MEK were lowly ranked targets compared to S6K and p38 (Ranking obtained from Table 1). We suggest that the ability of RAD51 to bind c/EBPβ can increase expression of key components of the signaling pathway. This is supported by the observation of RAD51-mediated changes in actin dynamics via p38 signaling to HSP27.

Several key proteins identified here likely mediate the molecular mechanisms of signaling that underlies our combination therapy. We found that RAD51 depletion and DNA damage activated the AKT pathway with enhanced phosphorylation of key proteins including STAT3 and AKT (Figure 6). The induction of signaling is likely enhanced by the KRAS mutation in MDA-MB-231 cells that results in constitutively activated PI3K signaling via mTOR to STAT3 [33]. As a mechanism of cell survival, STAT3 can induce transcription of specific target genes. We have previously established that RAD51 also contributes to changes in transcription via binding of the transcription factor c/EBPβ [12]. Interestingly STAT3 and c/EBPβ have overlapping consensus sequences in breast cancer promoters, suggesting competitive regulation [34]. Analysis of transcription after RAD51 depletion revealed loss of c/EBPβ transcript stability and p38 (a target gene of c/EBPβ). This scenario could result in the loss of c/EBPβ occupying promoters allowing STAT3 transcription and upregulation of kinase expression that result in the observed enhanced phosphorylation of STAT3.

Activation of DNA repair increases ERK1/2 phosphorylation [35] and p38 phosphorylation [36]. We observed significant ERK1 signaling in particularly when targeting RAD51 *in vitro* and both *in vitro* and *in vivo* under triple therapy. This might explain the ability of p38 inhibition to potentiate the effect of RAD51 and PARP inhibition. In the case of ERK1/2 phosphorylation after the triple combination, the difference in response observed between MDA-MB-231 cells (p53-mutant/BRCA1-wild type) and MDA-MB-436 cells (p53-low/BRCA1-mutant) may relate to BRCA status. A relation between BRCA1 and ERK1/2 during DNA damage response and cell cycle arrest has been previously described [35]. The different genetic context, particularly p53 status, of MDA-MB-436 compared to that of MDA-MB-231 could also attribute to the cell G2 arrest/mitotic catastrophe [36] in the p53-low MDA-MB-436 compared to apoptosis in the p53-mutant MDA-MB-231 cells. A function independent of signaling, ERK1 is capable of blocking the translocation of c/EBPβ localization to the nucleus via p38 in astrocytes [37]. In the absence of p38 signaling we suggest this function does not occur. Therefore, there may be no c/EBPβ transcriptional activity due to cytoplasmic retention via ERK1 and down regulation of transcript. This could explain the reduced transcript stability of c/EBPβ and p38 observed after RAD51 depletion and previous published observations for RAD51 depletion inhibiting c/EBPβ transcriptional activity [12].

In addition to RAD51 depletion enhancing p38 signaling, we observed depletion of phosphorylated-HSP27. p38 forms a complex with MK2 which can phosphorylate HSP27 in the presence of DNA damage [38]. Under the control of p38/MK2, phosphorylated-HSP27 releases its inhibitory activity on filament capping of actin polymers. This promotes polymerization and cell migration [39]. Thus, loss of HSP27 signaling promotes filament capping. We observed associated changes in cell morphology and migration after RAD51 depletion, which could be accounted for by loss of phosphorylated-HSP27 [12]. Depletion of HSP27 in human breast cancer cell line MDA-MB-231 reduces cell migration and invasion, and this attenuation of invasion correlates *in vivo* with a decreased ability of breast cancer cells to metastasize and grow in the skeleton [40]. In a comprehensive study it was shown that down-regulation of HSP27 in MDA-MB-436 breast cancer cells induced long-term dormancy *in vivo* [41] . Thus, the targeting of RAD51, PARPi and p38 also may have the ability to prevent metastatic dissemination of breast cancer cells and this forms part of our ongoing investigations.

The targeting of RAD51, PARP1 and p38 represents a potential effective therapy in TNBC that is not circumvented by “synthetic lethal resistance” observed via pressure on the DNA repair pathway. The unique combination utilizes a “pressure release valve” by targeting p38, which can drive survival by several described signaling mechanisms.

## MATERIALS AND METHODS

### Animal ethics

All animal procedures were conducted in accordance with Australian National Health and Medical Research regulations on the use and care of experimental animals, and approved by the QIMR Berghofer Medical Research Institute Animal Ethics Committee (approval P1301).

### Cell lines

TNBC MDA-MB-231, MDA-MB-436, and BT549 lines were acquired from ATCC, PMC42ET was a gift from Robin Anderson (Peter MacCallum Cancer Centre, Melbourne, Australia). BT549 was engineered for overexpression of RAD51 using Origene letinviral construct and empty vector as control (SKU: RG218333).

### Sensitivity to single DNA damaging and signaling inhibitor agents

MDA-MB-231, PMC42-ET and MDA-MB-436 breast cancer cell lines were analyzed for sensitivity to; PARPi (ABT-888-Veliparib), p38i (LY222882), DNA-PKi (KU-57788), S6K (PF 4708671), ATMi (KU-55933), PAKi (IPA3), Ligase IVi (SCR7), CHK1i (AZD7762), PYK2i (PF-562271), HSP90 (AUY922), GSK3i (CHIR-98014), DNA-PKi(2) (NU7026), MEKi(2) (AS703026), EGFRi (W2992-Afatinib), PDKi (GSK2334470), PKCi (LY317615), MEKi (AZD6244), PLCi (U73122), IGF-1R (GSK1904529A) and ERK5i (BIX 02189). All drugs were purchased from Selleck Chem. Each cell line was targeted with two different RAD51 siRNA or scrambled control siRNA and 1×10^4^ cells/well plated in the presence of 0-100 μM inhibitor and incubated for 120 hours. Growth inhibition was measured by MTS assay (Cell titer 96 AQueous Promega) measured at 500 nm. Data was analyzed using GraphPad Prism and plotted as nonlinear regression line of best fit for IC50. IC50 was then compared between non-targeted and RAD51 depleted cells and expressed as a ratio difference. The average between the three different cell lines provided the order of efficacy against TNBC.

### Combination therapy dose curves

Each of MDA-MB-231, MDAM-MB-436 or PMC42ET cell lines were incubated in the presence of each drug individually (0-100 μM) plus DMSO controls, as well as in combination with PARPi (2.5 μM ABT-888) or p38i (10 μM LY222882) and measured by MTS assay. The plots were standardized to DMSO untreated cells and represent line of best-fit non-linear regression +/−SEM.

### Kinome array

We performed the Proteome Profiler^™^ arrays as per our previous studies [18] and manufacturers' instructions. In summary, cells were washed with cold PBS and collected by trypsinization, lysates from breast cancer cell lines were prepared using the lysis buffer provided in the kit. Lysates were then hybridized to the arrays as per manufacturer instructions (R&D Systems, MN, USA). Phosphorylation levels were detected with the FujiFilm LAS-3000 Imaging System (Fuji Photo Film, Tokyo, Japan) using Millipore Immobilon Western chemiluminescent HRP substrate. Protein band intensity was determined using the “Protein array analyser” quantitation function of Image J v 1.43.

### Flow cytometry analysis

Cultures were trypsinized to single cell suspensions, washed with phosphate-buffered saline (PBS)/10% FBS, fixed with 70% cold ethanol and stained with propidium iodide containing RNAse. Flow cytometry analysis was performed on 10,000 gated events per sample. Cells were analysed and gated per subG1, G1, S, G2 and >G2 (Super G2-polyploid) populations.

### Long-term colony assays

After incubation with appropriate drug combinations, 1×10^3^ cells were plated per well in a 6-well plate and incubated with DMEM supplemented with 10% FBS and antibiotics. After 14 days incubation the colonies were washed with PBS, fixed with 70% ethanol and stained with 0.5% crystal violet in PBS for 5 minutes. Excess stain was removed with running water and plates scanned and quantitated using Image J software (NIH). Plots represent the mean and standard error of the mean (SEM) of 4 independent experiments.

### Immunoblotting

Protein lysates were prepared via whole cell lysis in ice-cold lysis buffer (150 mM NaCl, 10 mM Tris-Cl pH 7.4, 5 mM EDTA, 1% Triton X-100) supplemented with protease inhibitors (Leupeptin, Pepstatin and PMSF, Sigma Aldridge) as previously described [42]. Immunoblots were probed with anti-RAD51 (Santa Cruz Biotech), anti-p38, anti-phospho-p38 (Cell Signaling), anti-PARP (BD Biosciences), anti-MK2 (Cell Signaling), anti-HSP27, anti-phospho-HSP27 (Cell Signaling), anti-ERK1/2, anti-phospho-ERK1/2 (Cell Signaling) and anti-Δ-Actin, (Sigma) as a loading control. Membranes were developed using fluorescent-labeled secondary antibodies and visualized using the Odyssey system. Protein expression levels were determined by optical density versus actin loading controls using Image J software (NIH).

### Xenograft therapy

Cohorts of 5 week-old female nude NU-*Foxn1nu* or Balb/c mice with established 40 mm^3^ luciferase positive inguinal mammary fat pad tumors from 5×10^6^ cells of MDA-MB-231 were used. Mice were treated with 50 mg/kg B02 (Sigma), 25 mg/kg LY228820 (Selleck Chem) and 25mg/kg ABT-888 (Selleck Chem). Mice were treated via intraperitoneal injection. LY228820 and B02 were administered daily while ABT-888 was administered every second day. Tumor growth was monitored by caliper measurement and visualized using luciferase-mediated live animal imaging with the IVIS100 Xenogen system (Caliper Life Sciences).

### Expression analysis

RNA was isolated from RAD51 or c/EBPβ depleted MDA-MB-231 cells incubated in the presence or absence of 0.05mg/ml actinomycin D, using QIAGEN RNAEasy Mini Kit. cDNA was synthesized using RT^2^ First Strand Kit (QIAGEN). cDNA was analyzed on Roche Lightcycler 480 with validated primer sets (Supplementary Table S1). Fold expression change was calculated against actin and expressed as base-two exponential increase in RNA levels (2ΔΔCt)+/− SEM.

### Immunofluorescence

Approximately 5×10^4^ cells were seeded onto 18 mm glass coverslips. At 48 hours later, cells were gamma irradiated at 6 Gy. Coverslips were washed in (PBS), the cells were fixed in cold 70% ethanol and permeabilized in 0.5% Triton X-100 solution for 15 min at room temperature. Cells were blocked with 10% FBS in PBS and incubated with primary antibody (or alexa phalloidin) for 1 h and with secondary antibody for 30 min at room temperature. All antibodies were diluted in 5% FBS–PBS. Cells were then washed, counterstained with 4′,6′-diamidino-2-phenylindole (DAPI), and mounted. Primary antibody dilutions were as follows: Rad51, γH2Ax 1:1000 and 1:500 respectively. All secondary antibodies were used at 1:200.

### Statistical analysis

Results are presented as mean +/− SEM of replicate analyses and are either representative of or inclusive of at least three independent experiments. All statistical analyses were performed using two-tailed Student's *t*-tests in GraphPad PRISM 6 software. In all figures, significant differences between specified pair of conditions, as judged by *t*-test, are indicated using asterisks (*p<0.05; **p<0.01; ***p<0.005, ****p<0.001). IC50 doses for were calculated by interpolation of the sigmoidal dose response curves (Graphpad Prism 6.0 software).

## SUPPLEMENTARY FIGURES



## Abbreviations

TNBC: triple negative breast cancer
PARP1: poly(ADP-ribose)polymerase 1
p38: p38 Mitogen-Activated Protein Kinase
