# Supplementary material for: RAD51 inhibition in triple negative breast cancer cells is challenged by compensatory survival signaling and requires rational combination therapy
Source: Oncotarget. 2016 Aug 5;7(37):60087–100. doi: 10.18632/oncotarget.11065 (PMC5312370; doi:10.18632/oncotarget.11065)
Supplement: Supplementary file 1 [file oncotarget-07-60087-s001.pdf]

RAD51 inhibition in triple negative breast cancer cells is challenged by compensatory survival signaling and requires rational combination therapy

SUPPLEMENTARY FIGURES

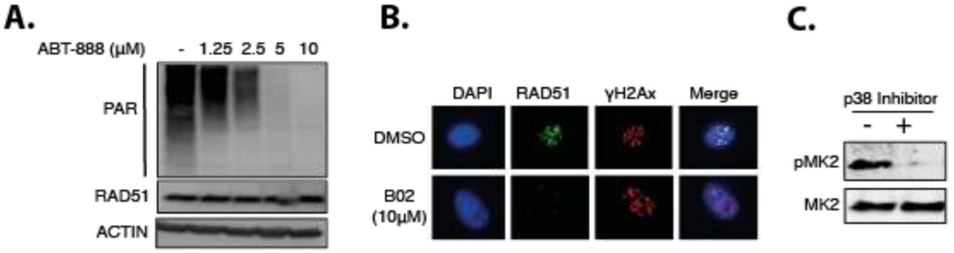

Supplementary Figure S1: The activity of the inhibitors were confirmed by **A.** Reduction in PARylated proteins in a dose dependent manner by ABT-888, **B.** Inhibition of RAD51 foci formation after irradiation by B02 and **C.** Inhibition of phosphorylation of the downstream target of p38MAPk, MK2 in the presence of 10µM LY2228820 in MDA-MB-231 cells.

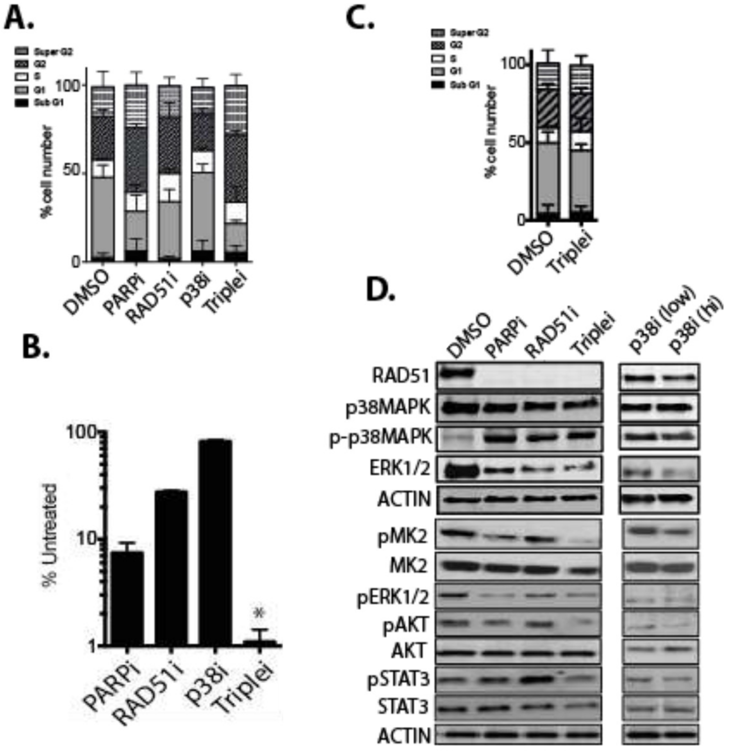

Supplementary Figure S2: **A.** Cell cycle profile analysis of MDA-MB-436 at 72h post treatment with individual drugs 10µM RAD51i, 10µM p38i and 2.5µM PARPi and triple combination, **B.** Colony formation after 14 days normalized against DMSO treated cells (**p=0.027). **C.** Cells treated with DMSO and triple combination were compared for cell cycle profile after 14 days colony formation. Cell cycle and colony formation were plotted as the mean of three independent experiments+/- SEM. **D.** Protein expression analysis on key signaling proteins after 72 hours treatment.

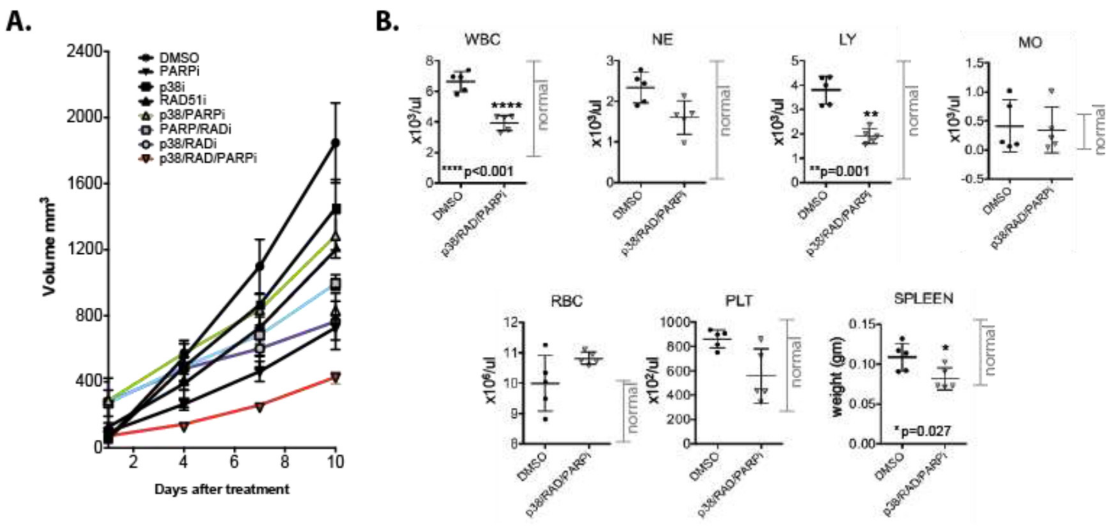

Supplementary Figure S3: A. Tumor burden plotted as volume in mm^3 for all single arm controls, double combinations and triple combination (+/- SEM) treatments with RAD51, PARP and p38 inhibitors. **B.** End point whole blood analysis of DMSO vs triple combination treated mice; WBC-white blood cell count, NE-neutrophils, LY-leukocytes (****p=0.001), MO-monocytes, RBC-red blood cells, PLT-platelets and SPN-spleen weight (*p=0.027).

A.

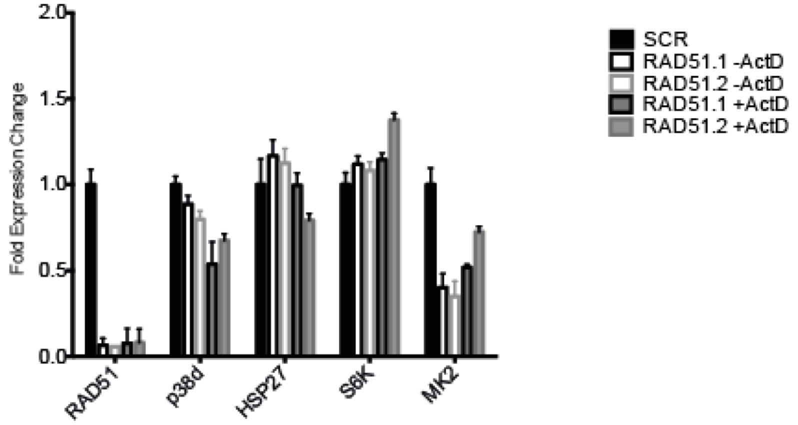

B.

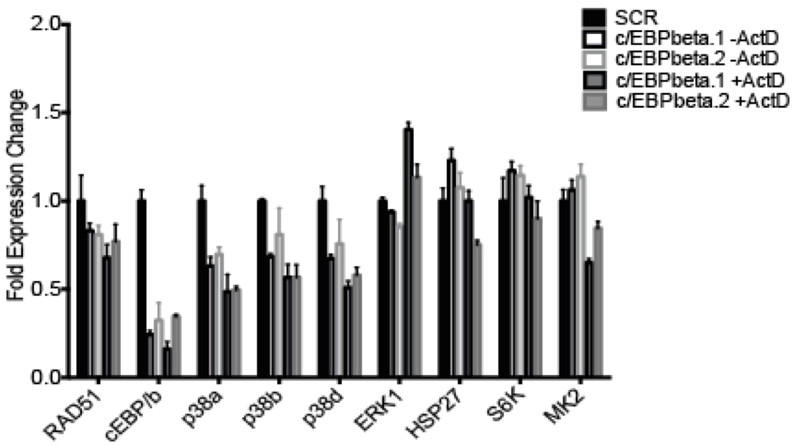

Supplementary Figure S4: A. mRNA expression analysis of key p38MAPk pathway genes after knockdown of RAD51 with two different targeting RNAi and **B.** mRNA expression analysis after targeting of c/EBPβ with two different targeting RNAi. The plots represent fold change in relation to GAPDH housekeeping gene and standardized to non-targeting control.
